# Comparison of Multi-Gene Testing Data Between Fresh and Formalin-Fixed Specimens From Core Needle Biopsy in Patients With NSCLC

**DOI:** 10.3389/pore.2021.1609931

**Published:** 2021-12-13

**Authors:** Zhi-Gang Yao, Zhi-Gang Wei, Xian-Kui Cheng, Guang-Hui Huang, Yuan-Yuan Zong, Min Meng, Jia-Mei Li, Xiao-Ying Han, Jia-Wen Xu, Jiao Wang, Hai-Yan Jing, Wen-Hong Li, Zhi-Xin Cao, Yang Ni, Xi-Chao Sun, Xia Yang, Xin Ye

**Affiliations:** ^1^ Department of Pathology, Shandong Provincial Hospital Affiliated to Shandong First Medical University, Jinan, China; ^2^ Department of Oncology, The First Affiliated Hospital of Shandong First Medical University & Shandong Provincial Qianfoshan Hospital, Shandong Key Laboratory of Rheumatic Disease and Translational Medicine, Shandong Lung Cancer Institute, Jinan, China; ^3^ Department of Oncology, Shandong Provincial Hospital Affiliated to Shandong First Medical University, Jinan, China

**Keywords:** pathology, EGFR, lung cancers, frozen section examination, fresh frozen tissue

## Abstract

**Purpose:** Currently, formalin-fixed paraffin-embedded (FFPE) tissue specimens are the conventional material for gene testing for non-small cell lung cancer (NSCLC) patients. In our study, we aimed to develop a quick gene testing procedure using fresh core needle biopsy samples from NSCLC patients.

**Methods:** In total, 77 fresh NSCLC samples obtained from core needle biopsy were evaluated by frozen section examination. If the NSCLC diagnosis and adequate tumor cell counts were confirmed by histopathology, the fresh tissues were used to extract DNA and subsequent gene testing by ARMS-PCR. Meanwhile, the paired FFPE core needle biopsy samples from 30 NSCLC patients also underwent gene testing.

**Results:** In total, 77 fresh samples showed an EGFR mutation rate of 61.0%, higher than the levels in the Asian. Following a comparison of gene testing results with fresh tissues and paired FFPE tissues from the 30 patients, no significant difference in the DNA concentration extracted from fresh tissues and FFPE tissues was found. However, DNA purity was significantly higher in fresh tissues than that in FFPE tissues. Gene testing detected the same gene mutations in 93.3% of cases in fresh tissues and paired FFPE tissues. The gene testing procedure using fresh biopsy samples greatly shortens the waiting time of patients.

**Conclusion:** The multi-gene mutation testing using fresh core needle biopsy samples from NSCLC patients is a reasonable, achievable, and quick approach. Fresh tissues may serve as a potential alternative to FFPE tissues for gene testing in NSCLC patients.

## Introduction

Patients with non-small cell lung cancer (NSCLC) often harbor driver mutations in multiple oncogenes, including EGFR, RAS, ALK, ROS1, BRAF, HER2, RET, etc., [1]. Gene mutation testing for lung cancer is important for identification of potentially efficacious targeted therapies. In particular, when it comes to patients with advanced NSCLC (stage III and IV), they have lost the opportunity of surgical therapy. As a result, gene mutations analysis is critical to developing the individualized clinical treatment strategy for those patients [1]. Currently, formalin-fixed paraffin-embedded (FFPE) tissue specimens are a conventional and valuable source of sample material for mutation analysis. FFPE tissues effectively preserve the cellular, architectural, and morphological details and allow easy storage at room temperature for extensive periods. Moreover, FFPE section examination will provide much quality-controlled data of samples, including a pathological diagnosis and tumor cell count. However, much effort has been made to verify whether fresh materials are suitable for molecular testing in recent years. It has been demonstrated that EGFR mutations can be detected using fresh samples obtained by CT-guided core needle biopsy [2]. Lai et al. also performed EGFR mutation analysis using surgically resected fresh specimens [3]. These screening procedures using fresh materials have proven to be achievable and cost-effective.

In this study, we evaluated the multi-gene mutation status in fresh tissues and paired FFPE tissues from core needle biopsy for target therapies of patients with NSCLC. Notably, a frozen section examination was involved in the quality control of fresh biopsy tissues, including the pathological diagnosis and tumor cell count. We reasoned that the comparable results in the two materials should validate the mutation analysis using fresh tissues from a core needle lung biopsy.

## Materials and Methods

### CT-Guided Percutaneous Needle Lung Biopsy

**FIGURE 1:**
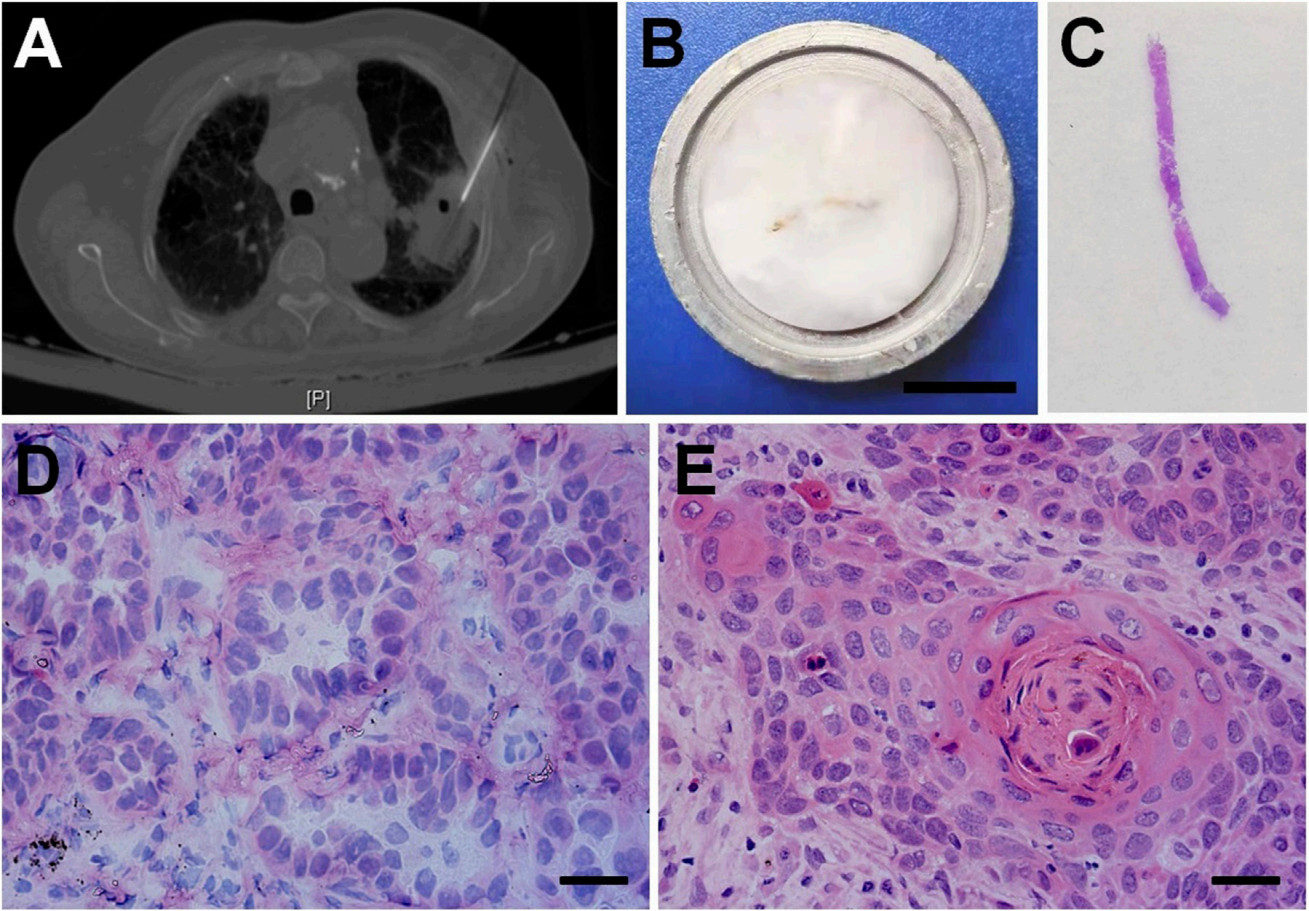
CT-guided core needle lung biopsy and the frozen section diagnosis of NSCLC. **(A)** CT-guided percutaneous needle biopsy for pulmonary nodules. **(B)** The fresh biopsy sample was embedded in gel medium on a metal tissue disc and then frozen rapidly to about −20°C. Bar = 1 cm. **(C)** The hemotoxylin-eosin (H&E) staining for a frozen tissue section. Histopathologic features of frozen section in the rapid diagnosis of lung adenocarcinoma **(D)**, squamous cell carcinoma **(E)**. Bars = 100 μm.

As previously described [4], 77 patients were placed in prone, supine, or lateral positions, whichever provided access to the best puncture pathway. All procedures were performed under CT (Neusoft CT, Neusoft Group Co., Ltd, China) guidance. A 16G sleeve-core needle (PRECISA fine-core needle, H. S. Hospital Service S. P. A, Aprilia, Italy) was pierced through the proximal edge of the lesion (Figure 1A). The core was then pulled out, with an 18G biopsy needle pierced into the lesion through the 16G sleeve. Each tumor was sampled 2–3 strips of samples, each measuring 1–1.5 cm. After the biopsy, the obtained materials were sent to the Department of Pathology for pathology examination.

### Pathology Examination

In the pathological laboratory, all experimental operations were carried out following standard biosecurity and institutional safety procedures. As shown in Figure 1B, the fresh biopsy samples were placed on a metal tissue disc and embedded in a gel medium (Kangleyi Inc. Jiangsu, China). The samples were then frozen rapidly to about −20°C (we consider them fresh frozen tissues, the same below). The 3-μm thick tissues were cut and stained with hemotoxylin-eosin (H&E) (Figure 1C). NSCLC was quickly diagnosed microscopically. For the patient with NSCLC, one strip of the entire fresh frozen (FF) tissues, which was rich in cancer cells (at least 200 cancer cells) examined by microscopy [5], was selected for DNA extraction. The rest of the strips were fixed in 10% formalin and prepared for paraffin sections and final pathology diagnosis. In our study, 30 patients with NSCLC confirmed by frozen section examination were enrolled to compare the results of multi-gene mutation testing using FF tissues and paired FFPE tissues.

### DNA Extraction

For FF tissues, DNA was extracted using the DNA kit (AmoyDx, Xiamen, China) according to the manufacturer’s instructions. For paired FFPE tissues, biopsy samples with at least 200 cancer cells were serially sliced on 8–10 slides with 5-μm thick and collected in specific tubes [5]. The tissue was deparaffinized and extracted DNA using a DNA FFPE tissues Kit (AmoyDx, Xiamen, China) according to the manufacturer’s instructions. The DNA purity extracted from FF tissues and FFPE tissues was measured with a NanoDrop spectrophotometer (Thermo Fisher Scientific, Waltham, MA, United States), and the OD260/280 ratio approximately equivalent to 1.8 was considered to be an ideal value.

### Multi-Gene Mutation Testing

According to the manufacturer’s protocol, amplification refractory mutation system (ARMS)-PCR was performed using AmoyDx Multi-Gene Mutations Detection Kit (AmoyDx, Xiamen, China) and AmoyDx MET Mutation Detection Kit (AmoyDx, Xiamen, China) to detect genes of EGFR, KRAS, BRAF, NRAS, HER2, and PIK3CA mutations, EML4-ALK, ROS1 and RET fusions, and MET Exon 14 skipping mutation.

### Efficiency of Gene Testing Using Fresh Tissues

In order to compare the efficiency of gene testing using FF and paired FFPE tissues, the time interval between physicians requesting and obtaining a test result was calculated.

### Statistical Analysis

We used SPSS 21.0 for statistical analysis. The differences between FF and paired FFPE tissues groups were analyzed using paired Student’s t-tests. Results were expressed as mean ± SEM. *p* < 0.05 was considered to be statistically significant.

## Results

### The Frozen Section Examination of NSCLC

**FIGURE 2:**
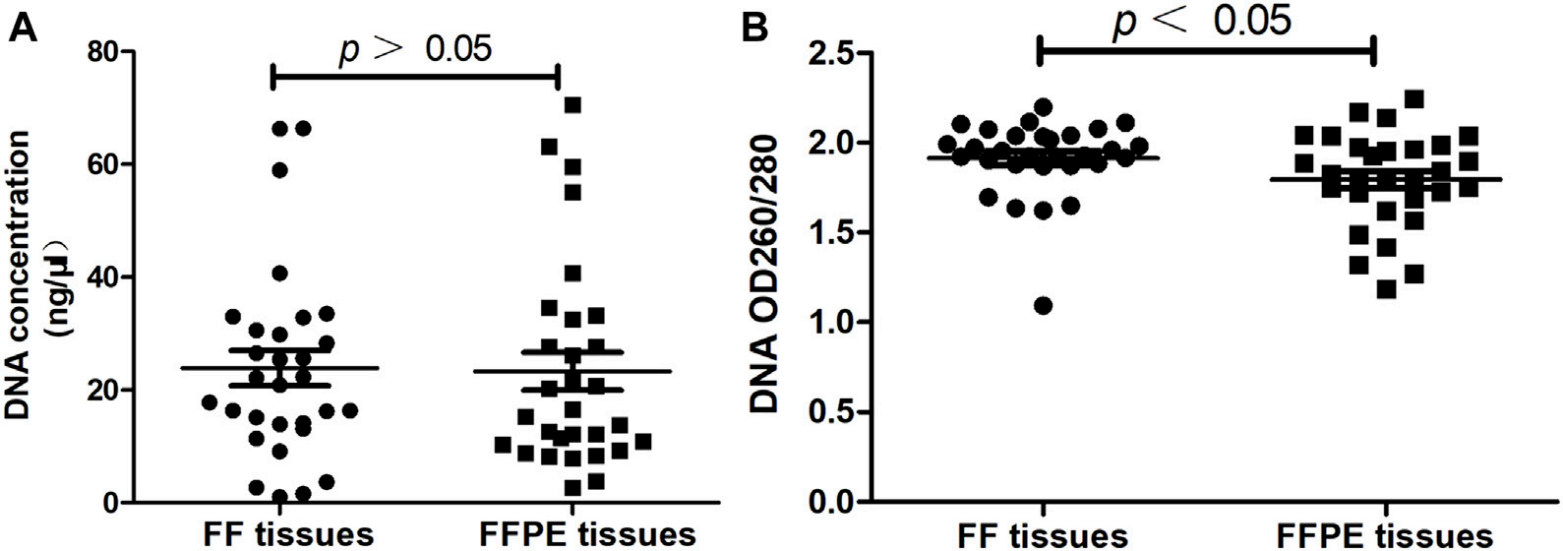
Evaluating the quality of DNA extracted from FF tissues and paired FFPE tissues (*n* = 30). **(A)** The concentration of DNA. **(B)** The purity of DNA.

The percutaneous CT-guided core needle biopsy is widely accepted as an accurate and safe procedure for the characterization of solid lung nodules (Figure 1A). The fresh biopsy samples were firstly dedicated to frozen section examination (Figures 1B,C). Pathologists were able to correctly identify lung adenocarcinoma (AC, Figure 2D) and squamous cell carcinoma (SCC, Figure 2E) with typical histological characteristics.

### Clinicopathological Characteristics of Patients

As shown in Table 1, frozen section examination confirmed the NSCLC diagnosis and adequate tumor cell count in the fresh tissues from 77 patients, which consisted of 39 males (50.6%) and 38 females (49.4%) with a median age of 65 years (range, 42–85 years) of which 32 were smokers (41.6%) vs. 45 non-smokers (58.4%). Frozen section examination revealed 70 (90.9%) AC, 6 (7.8%) SCC, and 1 (1.3%) adenosquamous carcinoma (ASC), which is consistent with the final pathological diagnosis using FFPE tissues. CT scanners displayed the masses mainly located in the superior lobe of the right lung (44.2%) and the superior lobe of the left lung (24.7%) with an average length of 3.8 cm and an average width of 2.8 cm. According to the eighth edition of the TNM Classification of lung cancer, most NSCLC patients were in stage IV (68.8%) followed by stage III (20.8%).

**TABLE 1:** The clinicopathological characteristics of 77 patients.

Characteristics	No. of patients (total, 77)	Frequency (%)
Age (y)		
≥60	51	66.2
<60	26	33.8
Gender		
Male	39	50.6
Female	38	49.4
Smoking		
Yes	32	41.6
No	45	58.4
Location		
Right upper lobe	34	44.2
Left upper lobe	19	24.7
Right lower lobe	13	16.9
Left lower lobe	6	7.8
Right Middle lobe	3	3.9
Left hilum	2	2.6
TNM stage		
IV	53	68.8
III	16	20.8
II	5	6.5
I	3	3.9
Pathological diagnosis		
AC	70	90.9
SCC	6	7.8
ASC	1	1.3

Abbreviations: AC, adenocarcinoma; ASC, adenosquamous carcinoma; SCC, squamous cell carcinoma.

To evaluate the difference in mutation testing between FF tissues and FFPE tissues, 30 patients with NSCLC in stage IV were randomly selected in our study. As listed in Table 2, the 30 patients consisted of 13 (43.3%) males and 17 (56.7%) females with a median age of 62.5 years (range 43–74 years). In total, 27 (90.0%) AC and 3 (10.0%) SCC were confirmed by frozen section examinations. The final pathological diagnosis in paraffin sections was all in accordance with the frozen section diagnosis. All patients had lost the opportunity of surgical treatment due to extrathoracic metastases.

**TABLE 2:** The gene mutation results of 30 enrolled patients.

Case No.	Gender	Age	FF tissues		FFPE tissues	
			FS Dx.	Variant analysis	PS Dx.	Variant analysis
1	F	74	AC	L858R and HER2	AC	L858R
2	F	51	AC	L858R	AC	L858R
3	F	75	AC	HER2	AC	HER2
4	F	55	AC	19-del	AC	19-del
5	M	43	AC	Wild type	AC	Wild type
6	F	63	AC	KRAS	AC	KRAS
7	F	68	AC	19-del and L858R	AC	19-del
8	M	62	AC	L858R	AC	L858R
9	F	53	AC	19-del	AC	19-del
10	F	65	AC	L858R	AC	L858R
11	F	46	AC	L858R	AC	L858R
12	M	82	SCC	NRAS3	SCC	NRAS3
13	F	69	AC	L858R	AC	L858R
14	F	66	AC	L858R	AC	L858R
15	F	65	AC	L858R	AC	L858R
16	M	51	AC	19-del	AC	19-del
17	M	70	AC	Wild type	AC	Wild type
18	F	60	AC	19-del	AC	19-del
19	M	63	AC	Wild type	AC	20-ins
20	F	51	AC	19-del	AC	19-del
21	M	71	AC	L858R	AC	L858R
22	M	70	AC	19-del	AC	19-del
23	F	58	AC	L858R	AC	L858R
24	M	75	SCC	PIK3CA	SCC	Wild type
25	M	60	AC	L858R	AC	L858R
26	M	72	AC	Wild type	AC	Wild type
27	F	55	SCC	L858R	SCC	L858R
28	M	49	AC	19-del	AC	19-del
29	M	49	AC	19-del	AC	19-del
30	F	53	AC	L858R	AC	L858R

Abbreviations: AC, adenocarcinoma; Dx., diagnosis; F, female; FF, fresh frozen; FFPE, formalin-fixed paraffin-embedded; FS, frozen section; M, male; No., number; PS, paraffin section; SCC, squamous cell carcinoma.

### DNA Quality Assessment

The concentration and purity of extracted DNA from FF tissues and FFPE tissues were evaluated before the ARMS-PCR assay. Results showed there was no significant difference in the DNA concentration extracted from FF tissues and FFPE tissues (23.86 ± 3.10 vs. 24.07 ± 3.27, *p* > 0.05, Figure 2A). As for DNA OD260/280 ratio, results showed DNA purity was significantly higher in FF tissues than that in FFPE tissues (1.91 ± 0.04 vs. 1.80 ± 0.05, *p* < 0.05, Figure 2B).

### Gene Mutation Testing

Among the 77 patients with NSCLC, 15 (19.5%) patients displayed the wild-type status of genes. In total, 47 (61.0%) patients carried EGFR mutations, including 33.8% EGFR L858R mutation and 28.6% EGFR 19-del mutation (Table 1). In contrast, a subset of patients displayed KRAS (7.8%), HER2 (5.2%), BRAF (2.6%), and ROS1 (2.6%) mutations. In addition, 5 (6.5%) patients harbored concomitant mutations in one or two of these genes.

**FIGURE 3:**
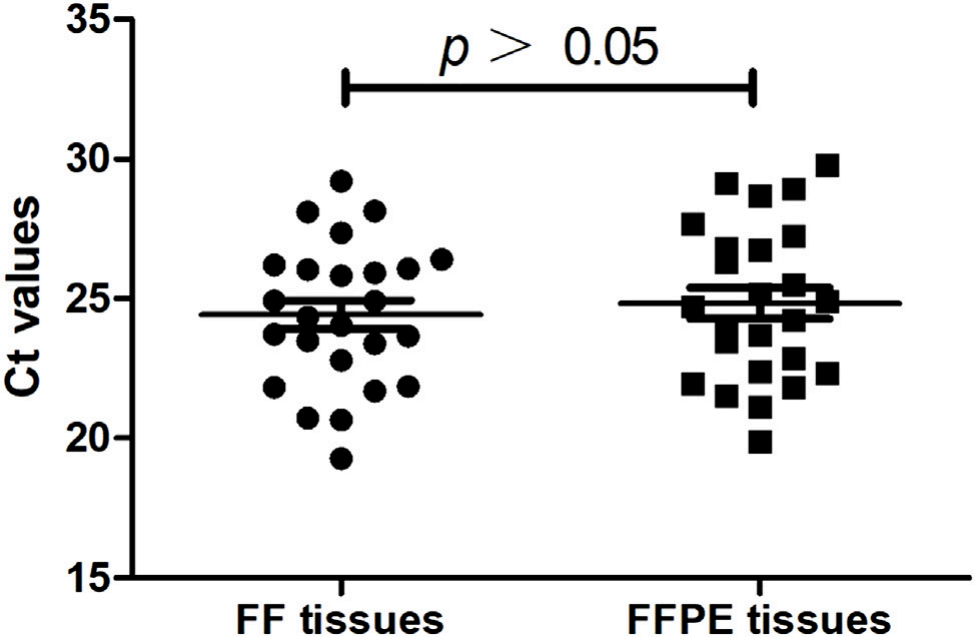
The cycle threshold (Ct) value of the PCR reaction between the systems using FF tissues and paired FFPE tissues (*n* = 30).

**FIGURE 4:**
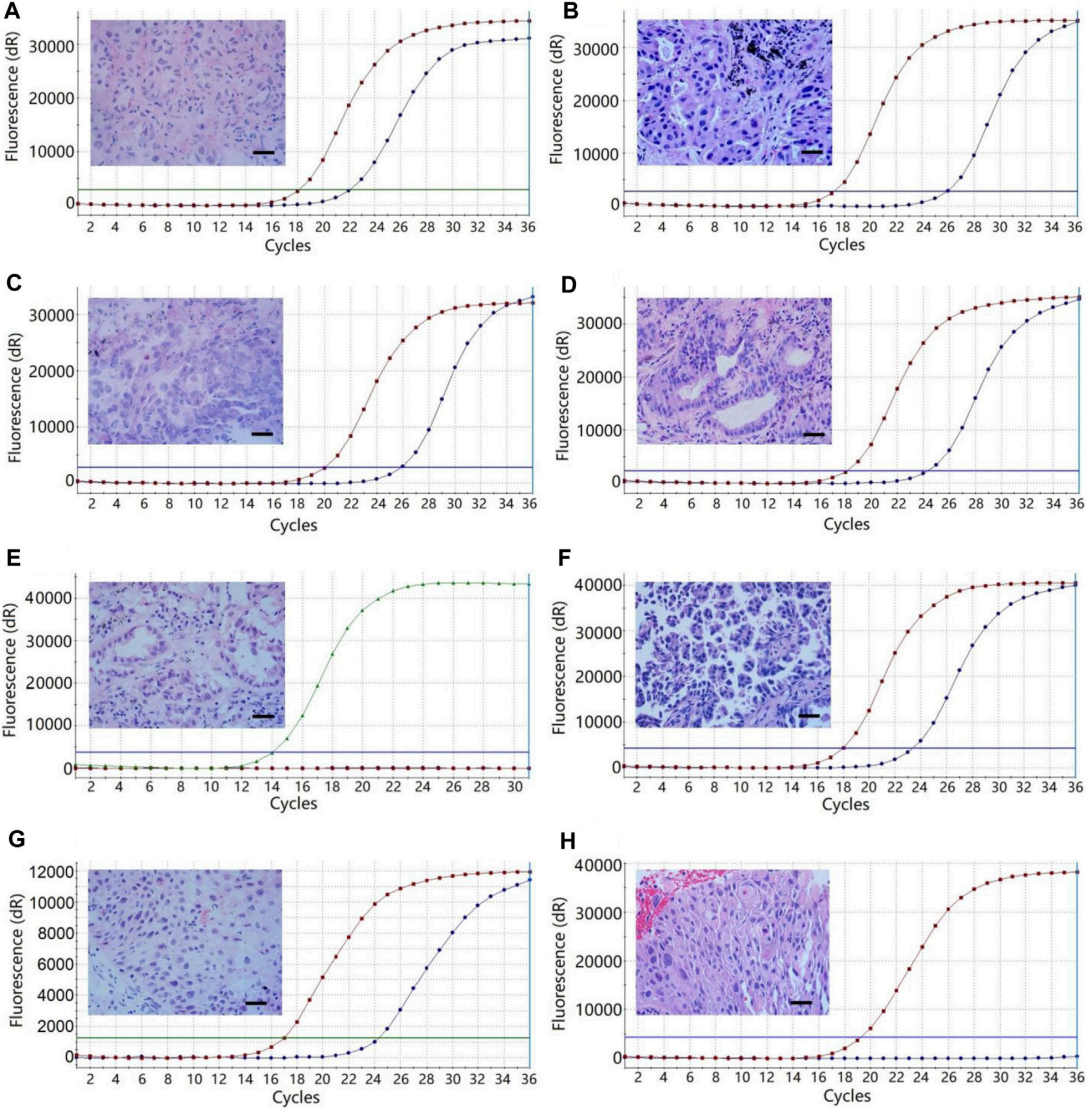
The multi-gene mutation results of 30 patients using FF tissues and paired FFPE tissues. **(A)** Case 1, HER2 mutation of the lung adenocarcinoma with a predominant acinar pattern in FF tissue. **(B)** Case 1, EGFR L858R mutation of the lung adenocarcinoma with an acinar pattern in paired FFPE tissue. **(C)** Case 7, EGFR L858R mutation of the lung adenocarcinoma with a predominant acinar pattern in FF tissue. **(D)**, Case 7, EGFR 19-del mutation of the lung adenocarcinoma with an acinar pattern in paired FFPE tissue. **(E)** Case 19, wild-type status of the lung adenocarcinoma with a predominant acinar pattern in FF tissue. **(F)** Case 19, EGFR 20-ins of the lung adenocarcinoma with a predominant micropapillary pattern. **(G)** Case 24, PIK3CA mutation of the squamous cell carcinoma in FF tissue. **(H)** Case 24, wild-type status of the squamous cell carcinoma in FFPE tissue. Bars = 100 μm. The red and green curves represent the positive controls, while the blue curves represent the tissue samples of NSCLC.

Gene mutation testing was evaluated in 30 patients using FF tissues and paired FFPE tissues. We compared the cycle threshold (Ct) value of the PCR reaction between the systems using FF tissues and paired FFPE tissues. Results showed no significant difference in Ct value between the reactive systems using FF tissues and paired FFPE tissues (24.43 ± 0.51 vs. 24.83 ± 0.56, *p* > 0.05, Figure 3). We detected the same gene mutations in 28 (93.3%) cases of FF tissues and paired FFPE tissues, including 13 (43.3%) cases of EGFR L858R, 9 (30.0%) cases of EGFR 19-del, 1 (3.3%) case of HER-2 mutation, 1 (3.3%) case of KRAS mutation, 1 (3.3%) case of NRAS mutation, and 3 (10.0%) cases of wild type. However, FF tissues in two cases of AC showed two gene mutations (Case 1: HER2, Figure 4A and EGFR L858R; Case 7: EGFR L858R, Figure 4C, and 19-del), while the paired FFPE tissues just displayed one gene mutation (Case 1: EGFR L858R, Figure 4B; Case 7: EGFR 19-del, Figure 4D). Histological examination showed a pure acinar pattern in the FF section and paired FFPE section (Figures 4A–D). Besides, FF tissues in two (13.3%) cases showed fully inconsistent gene variants compared with paired FFPE tissues. In detail, FF tissues showed wild-type status (Case 19: Figure 4E) in one case of AC, while the paired FFPE tissues showed EGFR 20-ins (Case 19: Figure 4F). Histological analysis showed a pure acinar pattern in the FF section (Figure 4E), while a predominant micropapillary pattern (Figure 4F) was observed in the FFPE section. In another case of SCC, FF tissues showed PIK3CA mutation (Case 24: Figure 4G), while the paired FFPE tissues showed wild-type status (Case 24: Figure 4H). However, no histological difference was observed in the FF section and paired FFPE section (Figures 4G,H).

### Efficiency of the Gene Testing Using Fresh Tissues

**FIGURE 5:**
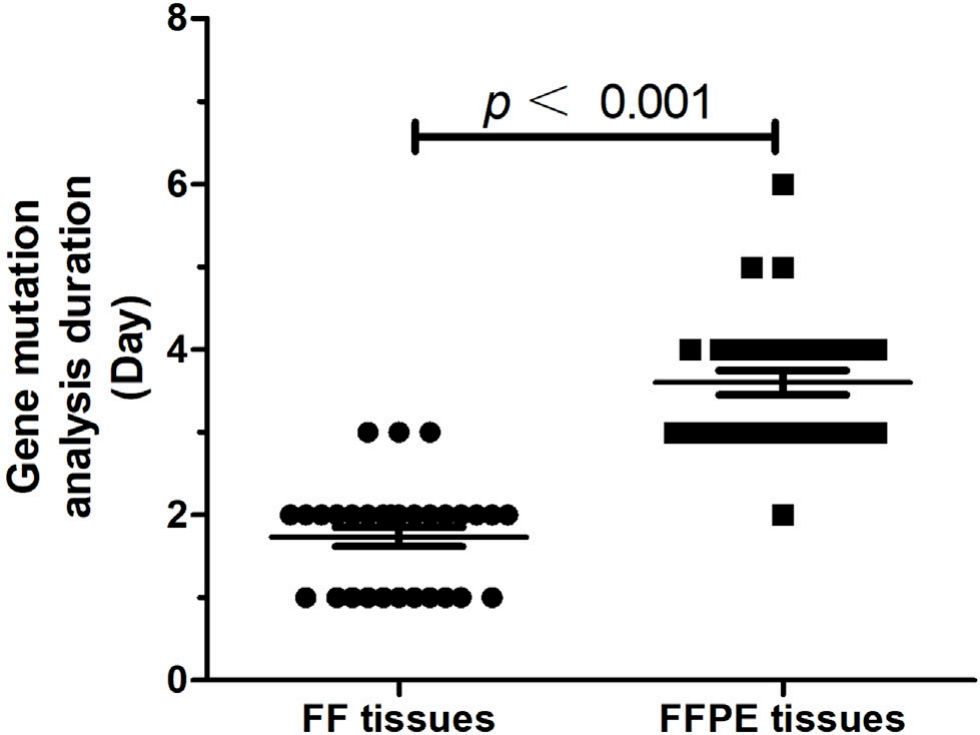
The time interval (day) between physicians requesting and obtaining a test result (*n* = 30).

In order to analyze the efficiency of gene testing, we measured the time interval between physicians requesting and obtaining a test result. Results showed gene mutations testing using FF tissues had a significantly shorter time interval (1.73 ± 0.12 days) as compared to that using FFPE tissues (3.60 ± 0.15 days, *p* < 0.001, Figure 5).

## Discussion

Although lung cancer is the second most commonly occurring cancer worldwide (11.4% of the newly diagnosed cancer cases), lung cancer is still the leading cause of cancer death (18.0% of total cancer deaths) according to the latest data released by the International Agency for Research on Cancer (IARC) [6]. In China, lung cancer is the most commonly diagnosed cancer and the leading cause of cancer death. In 2014, the age-standardized incidence rate of lung cancer was 36.71 per 100, 000, while the age-standardized mortality rate was 28.49 per 100, 000 [7]. NSCLC comprises 85% of lung cancer and the majority of these patients are at an advanced stage when diagnosed [8]. For a subgroup of advanced NSCLC patients with gene mutations, they could benefit from corresponding targeted therapies, presenting as tumor shrink, acceptable side-effect profile, and improved overall survival [9]. Therefore, the relevant consensus and guidelines in China have emphasized the necessity and importance of assessment on driver gene status, the standardization of experimental operations, the acquisition and processing of samples, and the selection of methodologies and scenarios of implementation [10].

Normally, the standard techniques of gene mutation testing in advanced NSCLC are based on polymerase chain reaction (PCR) and DNA sequencing using needle biopsy FFPE samples. The advantages of FFPE tissue samples include technical ease and low storage cost come at the expense of the sample quality. However, there are some disadvantages in DNA molecular analysis using FFPE tissues, including additional deparaffinization step, DNA fragmentation, and poor quality, resulting from formaldehyde-driven cross-linking between proteins and nucleic acids [11]. Meanwhile, the main advantage of fresh material is that DNA is in optimal conditions, free from possible artifacts of the fixation and paraffin-embedded processes. This improves the efficiency of amplification, without the need for DNA purification [12]. Recently, many studies evaluated the quality of gene mutation testing using fresh and FFPE samples. Suciu et al. demonstrated the validity of EGFR gene mutations from fresh tissues in lung adenocarcinoma patients [13]. Spencer et al. [14] compared the variants of 27 cancer-related genes between 16 pairs of FF and FFPE tissues from patients with lung carcinoma and found that the concordance rate was up to 96.8% in the single-nucleotide variants. In our study, the gene mutation results showed high concordance (93.3%) between FF tissues and paired FFPE tissues. There was no significant difference in DNA concentration between FF tissues and paired FFPE tissues. Moreover, the results of gene mutation analysis in our study demonstrated the concentration of DNA extracted from needle biopsy samples was adequate for multi-gene mutation testing. Indeed, Cheung et al. [15] reported that CT-guided core-needle biopsy of advanced NSCLC enables the acquisition of sufficient tissue for EGFR mutational analysis. In addition, no significant difference in Ct value was observed in ARMS-PCR systems between FF tissues and paired FFPE tissues. The Ct value of a reaction is defined as the cycle number when the fluorescence of a PCR product can be detected above the background signal. Therefore, the result of the study means the number of DNA templates was equal between FF tissues and paired FFPE tissues, though formaldehyde-driven cross-linking between proteins and nucleic acids.

In our study, 61.0% of patients carried EGFR mutations among 77 patients. According to a worldwide prospective study, Asian patients with lung adenocarcinoma showed 51.4% EGFR overall mutation frequency [16]. In the mainland of China, the overall EGFR mutation rate is 50.2% [17]. Our present study showed the overall mutation rate of EGFR was 61.0%, which is higher than the levels in the Asian and China mainland. Moreover, FF tissues in two cases revealed two gene mutations, while just one gene mutation was detected in the paired FFPE tissues. FF tissues from one case of SCC showed PIK3CA mutation, while the paired FFPE tissues showed wild-type status. However, histological heterogeneity was not found in FF tissues and paired FFPE tissues from the three cases. These results suggest gene testing using fresh tissues might increase gene mutations detection rates. We speculate that the fresh tissues from NSCLC seem to preserve more variants data than FFPE tissues. Indeed, Verhoest et al. [18] found only half of the true VHL abnormalities were identified in FFPE tissues as compared with that in FF tissues. However, Case 19 in our study showed wild-type status in FF tissues with pure acinar histology, while the paired FFPE tissues showed EGFR 20-ins with a predominant micropapillary pattern. This inconsistency can be attributed to the intratumor histological and genetic heterogeneity [19]. Micropapillary adenocarcinoma is considered equivalent to poorly differentiated adenocarcinoma and resembles morphology associated with high-grade lesions. Moreover, the EGFR-mutated biopsy samples from the advanced lung adenocarcinoma had a higher frequency of micropapillary pattern than EGFR-wild type tumors [20]. In spite of the potential problems of both sampling variation and tumor heterogeneity, our study shows that multi-gene mutation analysis using FF samples may provide valid, robust data.

**FIGURE 6:**
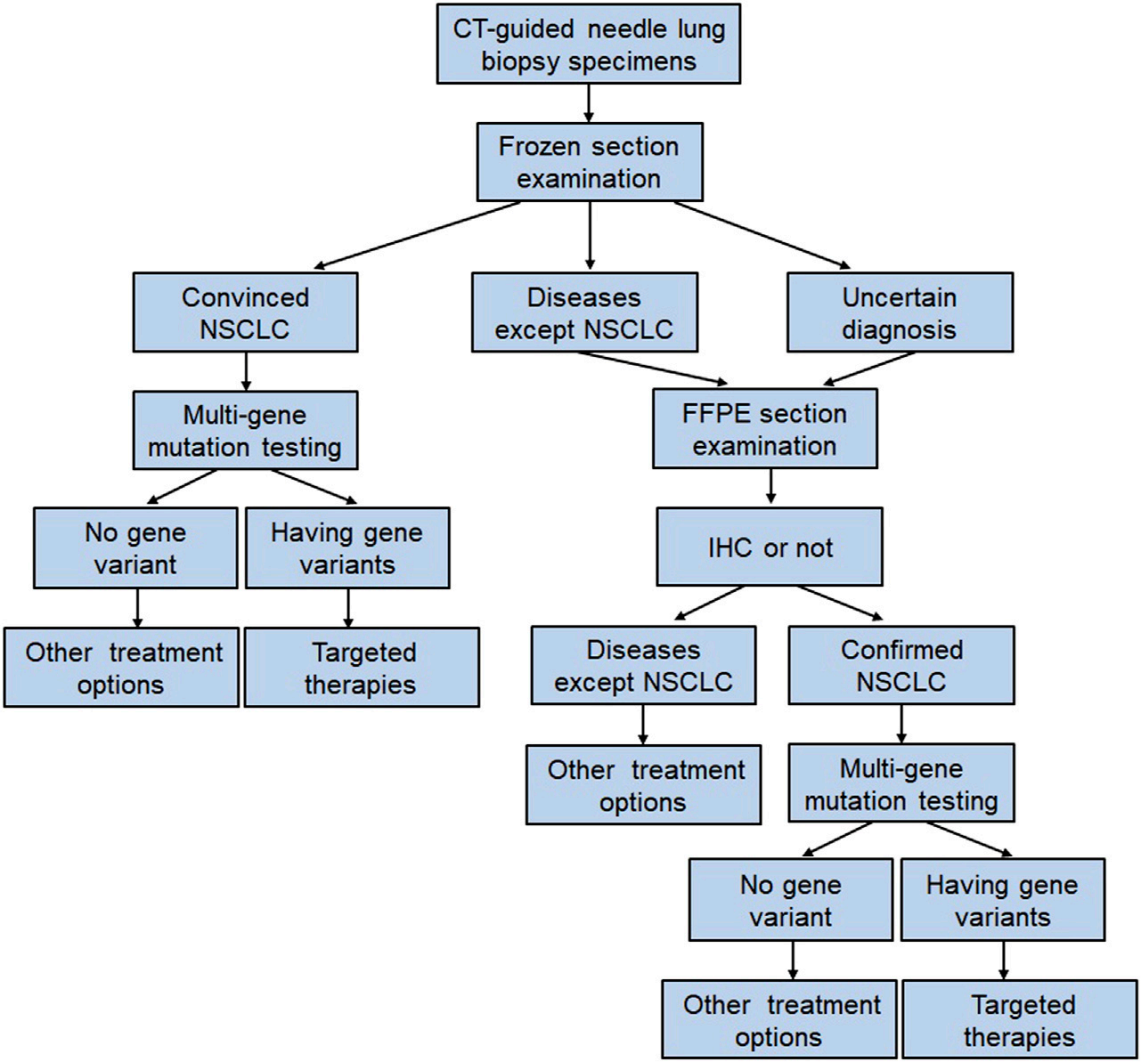
The multi-gene mutation testing procedure using CT-guided core needle lung biopsy samples. Abbreviations: FFPE, formalin-fixed paraffin-embedded; IHC, immunohistochemistry.

Although the fresh tissue has distinct advantages in gene analysis, the main limitation is that in fresh material, it is impossible to accurately evaluate the histological classification of tumor and estimate the tumor cell density, and this can lead to false-negative results. In our study, the frozen section examination is a rapid and reliable method for tumor diagnosis. It can accurately distinguish NSCLC from small cell carcinoma, sclerosing pneumocytoma, granulomatous lung disease, etc. Besides, the frozen section examination will also provide the data of tumor cell count to guarantee the success rate of gene analysis. Accordingly, we develop a procedure of gene mutation testing using fresh core needle biopsy specimens of NSCLC. As shown in Figure 6, samples from patients with solid pulmonary masses or suspected lung cancer were obtained by percutaneous CT-guided core needle biopsy. Frozen section examination using fresh biopsy samples quickly classified the masses as convinced NSCLC, diseases except for NSCLC, and uncertain diagnosis. The fresh samples diagnosed with convinced NSCLC were used to extract DNA and subsequent multi-gene mutation analysis. If NSCLC was not confirmed by frozen section examination, the fresh samples were fixed in 10% formalin and prepared for paraffin sections. Immunohistochemistry (IHC) was carried out when necessary. For example, an IHC panel that includes thyroid transcription factor-1 (TTF-1), Napsin A, CK5/6, p40, CD56, synaptophysin (Syn), and chromogranin (CgA) will be useful in the differentiation of the histological type of lung cancers [21]. The procedure will prevent misuse of molecular testing in lung cancer. If NSCLC was confirmed by final pathology diagnosis, FFPE tissues were used to extract DNA and multi-gene mutation analysis. The clinical treatment strategy for patients was optimized based on gene test results. Using this procedure of gene mutation testing, the time interval between physicians requesting and obtaining a test result has been shortened to fewer than 2 days. In Asian, the mean time interval for reporting the test was 17.6 days [17]. After all, timely treatment can delay progression and prolong the survival of patients with advanced NSCLC [22].

There are limitations to our study. First, there is a considerable risk of misdiagnosis during frozen section examination. We think pathologist experience is of crucial importance for the diagnostic accuracy of frozen sections. Moreover, the uncertain diagnosis of fresh tissues will not be allowed into the procedure of gene testing. In addition, careful clinical evaluation combined with pathology diagnosis may reduce the misdiagnosis of lung nodules. Second, in our study, the sensitivity and specificity of gene testing using FF tissues are 96 and 75% when compared with FFPE tissues. The high sensitivity and low specificity may be attributed to the selection of cases through frozen section examination. More cases are necessary to evaluate the specificity and sensitivity in future studies. However, the high coincidence rate (93.3%) of gene testing suggests a reliable detection procedure using FF tissues in our study.

In conclusion, we performed multi-gene mutation testing using fresh needle biopsy samples from patients with NSCLC. Our testing results indicated that: 1) It is feasible to detect multi-gene mutation using fresh needle biopsy samples of NSCLC. 2) Frozen section examination plays a critical role in the sample quality control, including pathological diagnosis of lung tumor and estimation of tumor cell count. 3) Fresh samples are more likely to increase the detection rate of gene mutations. 4) It greatly reduces the waiting time of patients, improves the efficiency of gene detection. In conclusion, our study demonstrates that multi-gene mutation testing using fresh core needle biopsy samples is a reasonable, achievable and quick approach. Fresh tissues from core needle biopsy serve as the potential alternative to FFPE tissues for multi-gene testing in patients with NSCLC.

## Data Availability

The original contributions presented in the study are included in the article/supplementary material, further inquiries can be directed to the corresponding authors.
